# Physical exercise as a treatment for persisting symptoms post-COVID infection: review of ongoing studies and prospective randomized controlled training study

**DOI:** 10.1007/s00392-023-02300-6

**Published:** 2023-09-12

**Authors:** Alexander Kogel, Moritz Machatschek, Ronja Scharschmidt, Carolin Wollny, Florian Lordick, Mohamed Ghanem, Ulrich Laufs, Sven Fikenzer

**Affiliations:** 1 Klinik und Poliklinik für Kardiologie, Universitätsklinikum Leipzig, Liebigstr. 20, 04103 Leipzig, Germany; 2 Zentrale Einrichtung für Physikalische Therapie und Rehabilitation, Universitätsklinikum Leipzig, Leipzig, Germany; 3 Medizinische Klinik und Poliklinik 2-Onkologie, Gastroenterologie, Hepatologie, Pneumologie, Infektiologie, Universitätsklinikum Leipzig, Leipzig, Germany; 4 Klinik und Poliklinik für Orthopädie, Unfallchirurgie und Plastische Chirurgie, Universitätsklinikum Leipzig, Leipzig, Germany

**Keywords:** Exercise, COVID-19, Fatigue, Post-COVID, Training

## Abstract

**Background and purpose:**

No evidence-based treatment is available for patients with persisting symptoms post-COVID-19 infection. We hypothesized that physical exercise may represent a safe and effective treatment option for post-COVID.

**Methods:**

We performed a systematic search of the literature that revealed a lack of randomized training studies in patients post-COVID. Based on these findings, a prospective randomized controlled study with open-label and blinded endpoint evaluation was designed. 272 patients with symptoms of fatigue persisting over 6 weeks post-COVID infection were screened. Patients with pathological cardiovascular findings were excluded. 57 patients consented and were randomized to 4 weeks of supervised personalized strength and endurance training or usual care. The follow-up period was 3 and 6 months.

**Results:**

There were no adverse events related to the training. Spiroergometry of the training group showed a significantly higher increase in VO2peak (10.0 ± 12.7% vs. 0.1 ± 8.9%, *p < *0.01, respectively) and oxygen pulse (9.8 ± 10.8% vs. 0.0 ± 13.9%, *p < *0.05, respectively). Parameters of the Multidimensional Fatigue Inventory-20, McGill Quality of Life Questionnaire, and Post-COVID-19 Functional Status were improved after 4 weeks in both groups. In the follow-up period, the total physical activity per week was significantly greater in the exercise group than in controls (1280 ± 1192 min vs. 644 ± 554 min, *p < *0.05, respectively). The improvements in fatigue and quality of life were not statistically different between the training and usual care groups.

**Conclusion:**

Exercise is safe and improves maximal exercise capacity in post-COVID patients. Fatigue and quality of life improve over time in individuals that are willing to participate in a training study irrespective of their allocation.

**Registration:**

German Clinical Trials Register: DRKS00026686. Date of registration: 27.09.2021.

**Graphical abstract:**

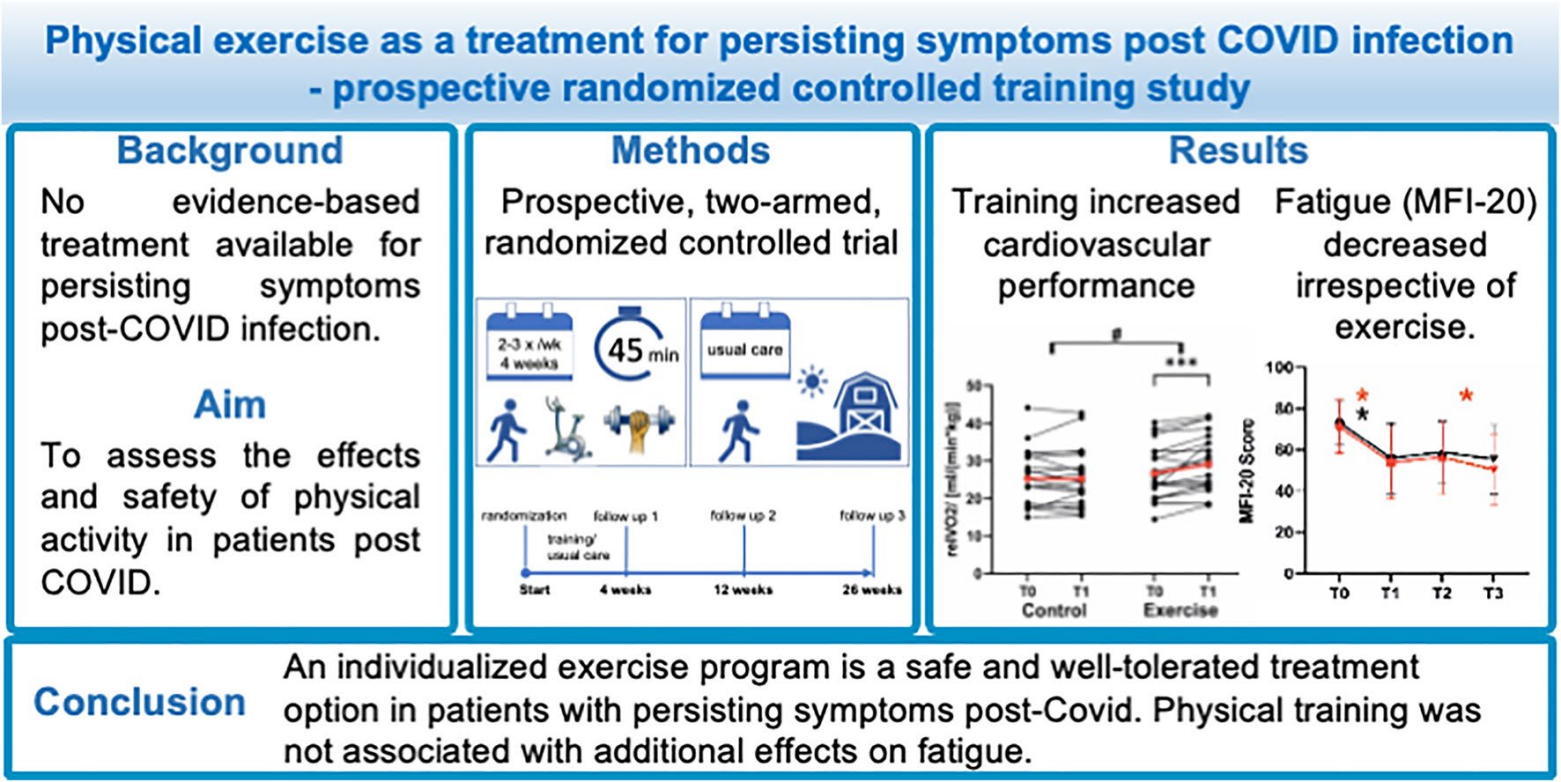

**Supplementary Information:**

The online version contains supplementary material available at 10.1007/s00392-023-02300-6.

## Introduction

Subsequent to the health problems of the acute phase of SARS-CoV-2 infection, the long-term consequences of SARS-CoV-2 infection are affecting large numbers of patients worldwide. Estimates of patients affected by persisting or new symptoms post-COVID reach from 6 to 28 percent of the infected population [1]. To date, no evidence-based treatment is available for persisting symptoms post-COVID infection. The most common symptoms post-COVID are fatigue, muscle weakness, and sleep difficulties [2]. Fatigue in particular is associated with a significantly reduced quality of life [3]. There is a strong overlap in the spectrum of the symptoms post-COVID and the chronic fatigue syndrome / myalgic encephalomyelitis, which is characterized by post-exertional fatigue and persistent symptoms related to cognitive and autonomous dysfunction [4]. Various triggers including viral infections (e.g. with the Epstein–Barr virus) are discussed for chronic fatigue syndrome [5].

The molecular mechanisms causing the symptoms post-COVID are not completely understood and are likely multi-factorial. A summary of these mechanisms is depicted in Supplemental Fig. 1. Physical exercise is well established to exert anti-inflammatory, vasculo-protective, and anti-aging effects [6–8].

Based on the potential mechanisms contributing to the persisting symptoms post-COVID and the potential effects of physical exercise on these pathologies we hypothesized that physical training may be associated with beneficial effects for patients with persisting symptoms post-COVID. However, in addition to the assessment of potentially positive effects, it is important to establish the safety of exercise post-COVID. Physical exercise has negative effects in mice with myocarditis [9]. In athletes, myocarditis is the underlying cause of sudden cardiac death in about 10 percent of cases [10].

We, therefore, performed a systematic search of the literature for training studies in patients post-COVID to review the available information on the safety and efficacy of physical exercise for these patients. The systematic review of the literature revealed a shortage of prospective, randomized training studies and a lack of information on safety. Therefore, we designed and performed a prospective study to test the effects of supervised personalized strength endurance training for 4 weeks or usual care on safety, fatigue, and quality of life.

## Methods

### Data source and search strategy

ClinicalTrials.gov, Deutsches Studienregister, PubMed, and the World Health Organization International Clinical Trials Registry Platform were searched to identify RCTs investigating exercise interventions in post-COVID syndrome. The date of the last search was February 16^th^, 2022. We used “exercise” in combination with “fatigue” or “post-COVID” as keywords and filtered for randomized controlled trials. Criteria for inclusion in this review were RCT design with an exercise intervention and a control group that underwent no intervention. The flow diagram of the screening is depicted in Supplemental Fig. 2.

### Study design

The post-COVID-training study (DRKS00026686**)** was approved by the Ethics Committee of the Medical Faculty, University of Leipzig (reference number 357/21-ek). Written informed consent was obtained from all the participants. This was a prospective, two-armed, randomized controlled trial. The primary outcome is the subjective improvement in perceived fatigue according to MFI-20 in at least 2 of 5 categories at follow-up (1/3/6 month-follow-up). Secondary outcomes are changes in spiroergometric parameters and strength (1 month-follow-up) and improvement in the McGill Quality of Life Questionnaire and Post-COVID Functional Status Scale (1/3/6 month-follow-up). The sample size was calculated to achieve a significant training effect measured as an improvement in VO2peak.

### Patient selection

**Fig. 1:**
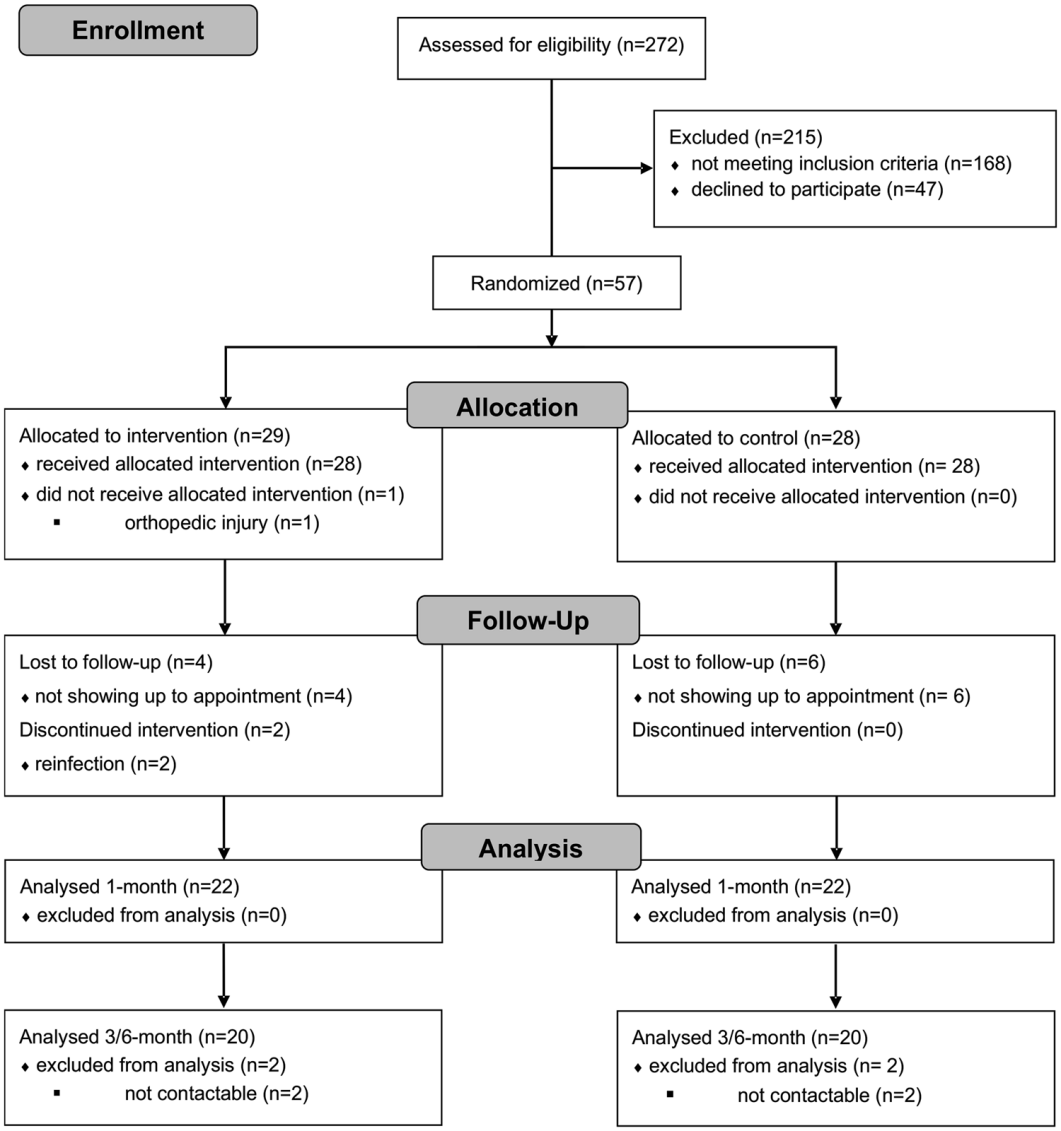
Consort diagram of the inclusion process in the PCTS study

Participants were recruited over 12 months through a post-COVID clinic at the Department of Internal Medicine, University Hospital Leipzig. Patients over the age of 18 with sustained fatigue symptoms (> 50 points with four or more dimensions affected on the MFI-20-questionnaire) at a minimum of 6 weeks after a COVID-19 infection with no known cardiac or pulmonary condition were eligible. The definition of “post-COVID syndrome” was defined in a consensus paper in late 2021 [11]. Our study was started before the publication of this consensus definition. In our study, four patients—two in each group—do not meet the new definition. Thus, over 90% of the study population fulfills the criteria of the “post-COVID syndrome”. Exclusion criteria entailed known heart failure with reduced or preserved ejection fraction, coronary artery disease, myocarditis, obstructive or restrictive pulmonary disease, or COVID-19-associated cardiovascular complications (e.g. pulmonary artery embolism, myocardial infarction, pulmonary fibrosis). The flow diagram of the inclusion into the study is shown in Fig. 1.

### Randomization

Participants were randomly allocated to the intervention (4 weeks of two to three times weekly personalized strength endurance training) or the control using a 1:1 ratio. The allocation sequence was produced via computer-generated random numbers. It was not possible to blind the participants or medical staff to the group allocation. Staff blinded to group allocation performed data entry and analysis.

### Examinations

All participants underwent a basic medical examination confirming eligibility.

### Intervention

Participation took place over 6 months comprising one assessment visit at baseline, and one visit at the end of the intervention, and two follow-up assessments after 3 and 6 months.

Participants randomly assigned to the intervention group were asked to attend twice- to thrice-weekly supervised 45-min exercise sessions consisting of strength and endurance exercises. Strength exercises were set for 70% of the before assessed one repetition maximum (1-RM) during isotonic concentric activity and an additional 20% overload during eccentric activity and included leg press, squats, pull, crunch, back extension, and press for 1 min and 30 s pause in between for two rounds. Endurance exercises were set to power in Watts at the before-assessed respiratory compensation point (RCP) with 60–70 revolutions per minute (rpm) and included using a bike-ergometer and crosstrainer. Exercise compliance was assessed using the Technogym assessment tool (Technogym™, TECHNOGYM GERMANY GMBH, Neu-Isenburg, Germany).

Participants randomly assigned to the control group were given no restrictions.

### Questionnaires

For all assessments, self-reporting questionnaires in German were used to assess fatigue (Multidimensional Fatigue Inventory-20) [12], quality of life (McGill Quality of Life Questionnaire (MQOL) [13], functional status (Post-COVID-19 Functional Status (PCFS) [14], and physical activity (WHO Global Physical Activity Questionnaire (GPAQ) [15].

### Strength measurements and cardiopulmonary exercise testing

Strength was evaluated using one repetition maximum (1-RM) of each aforementioned exercise. In addition, at each visit cardiorespiratory fitness was assessed using spirometry (Vyntus™ CPX, Vyaire Germany, Hoechberg, Germany) and a cardiopulmonary exercise test (CPET); performed on a semi-recumbent ergometer (GE eBike, GE Healthcare GmbH, Solingen, Germany) at a constant speed of 55–70 revolutions per minute (rpm) beginning at a workload of 25 W ramping 15 W per minute until voluntary exhaustion occurred. Each participant continued for an additional 5-min recovery period at a workload of 25 W. In the CPET, spirometry data were collected using a digital spirometer (Vyntus™ CPX, Vyaire Germany, Hoechberg, Germany). Maximum oxygen consumption (VO2max), minute ventilation (VE), and HR (GE-Cardiosoft, GE Healthcare GmbH, Solingen, Germany) were monitored continuously.

Safety of exercise was defined as no new symptoms or worsening of symptoms, no palpitations, no hospitalizations, and no clinical signs of heart failure.

### Statistical analysis

All statistical analyses were performed using GraphPad Prism 9 (version 9.4.1, GraphPad Software LLC), and Microsoft Office Excel (version 16.53, Microsoft). Continuous variables were expressed as mean value ± standard deviation (SD). Changes between two visits in one group were analysed using a paired *t* test. Group effects were analysed by unpaired t tests. Statistical significance was accepted for *p* value < 0.05. We used the CONSORT reporting guidelines [16].

## Results

### Prospective training study

57 patients with persisting symptoms post-COVID infection consented to participation and were randomized. The study took place from September 2021 to March 2023. The study ended after randomizing the targeted sample size. None of the patients needed hospital admission during the acute infection with SARS-CoV2. The clinical characteristics including echocardiographic and laboratory data are presented in Table 1. The mean age was 42.7 ± 13.4 years and 61% (*N = *27) were females.

**Table 1:** Baseline characteristics of the study population

Study population		Exercise	Control	*p*
Total	*N*	22	22	
Sex	(f;m)	13;9	14;8	
Age	y	40.4 ± 13.1	43.6 ± 14.3	0.275
Height	cm	172.7 ± 9.5	170.8 ± 12.2	0.584
Weight	kg	71.1 ± 10.4	75.1 ± 17.0	0.369
BMI	index	23.8 ± 2.7	25.5 ± 3.9	0.117
Training units/wks	number	2.3 ± 0.4		
Exercise compliance	%	96.7 ± 2.0		
Time post-COVID	days	284.5 ± 148.6	270.4 ± 151	0.761
LVEF	%	65.0 ± 5.6	65.3 ± 6.5	0.865
GLS	%	− 20.0 ± 1.5	− 20.0 ± 2.2	0.971
Laboratory data				
Hemoglobin	mmol/l	8.7 ± 0.7	8.6 ± 0.8	0.720
hsCRP	mmol/l	1.4 ± 1.4	1.4 ± 1.2	0.993
Troponin-T	pg/ml	4.3 ± 1.4	4.5 ± 1.9	0.777
NT-proBNP	pg/ml	71.6 ± 29.8	83.7 ± 62.4	0.450
Ferritin	ng/ml	121.9 ± 107.6	139.3 ± 109.5	0.645
Transferrin	g/l	2.7 ± 0.6	2.6 ± 0.3	0.608
TSH	mU/l	1.6 ± 0.6	1.4 ± 0.9	0.350

*BMI* body-mass index; *wks* weeks

We found no abnormalities or differences between the groups in echocardiographic measurements, laboratory data (Table 1), or spirometry (Table 2).

**Table 2:** Spirometry

		Exercise							Control							Group effects t1
		*t*0			*t*1			*p*	*t*0			*t*1			*p*	*p*
Spirometry																
FVC	l	4.2	±	1.1	4.2	±	1.1	0.767	4.1	±	1.0	4.1	±	1.0	0.748	0.845
FEV1	l	3.5	±	0.9	3.5	±	0.9	0.482	3.4	±	0.8	3.4	±	0.8	0.925	0.826
Tiffeneau	%	83.4	±	7.0	82.7	±	5.9	0.401	82.7	±	5.6	82.8	±	6.1	0.872	0.921
PEF	l/s	7.4	±	1.7	7.7	±	1.7	**0.036**	7.1	±	2.0	7.2	±	1.7	0.457	0.932

*FVC* forced vital capacity; *FEV1* forced expiratory volume in the first second; *PEF* peak expiratory flow

*p*-value < 0.05 are written bold

The patients of the exercise group underwent 9.2 ± 1.6 physical training sessions (7–12). The individualized combined training was very well tolerated by the participants and the exercise compliance was high at 96.7 ± 2.0%. There were no adverse events during the training sessions. The total physical activity (GPAQ) did not change over time (exercise pre: 1472 ± 1213 min/wk, post: 1518 ± 1623 min/wk, *p = *0.903; control pre: 1185 ± 1268 min/wk, post: 895 ± 888 min/wk, *p = *0.221).

**Fig. 2:**
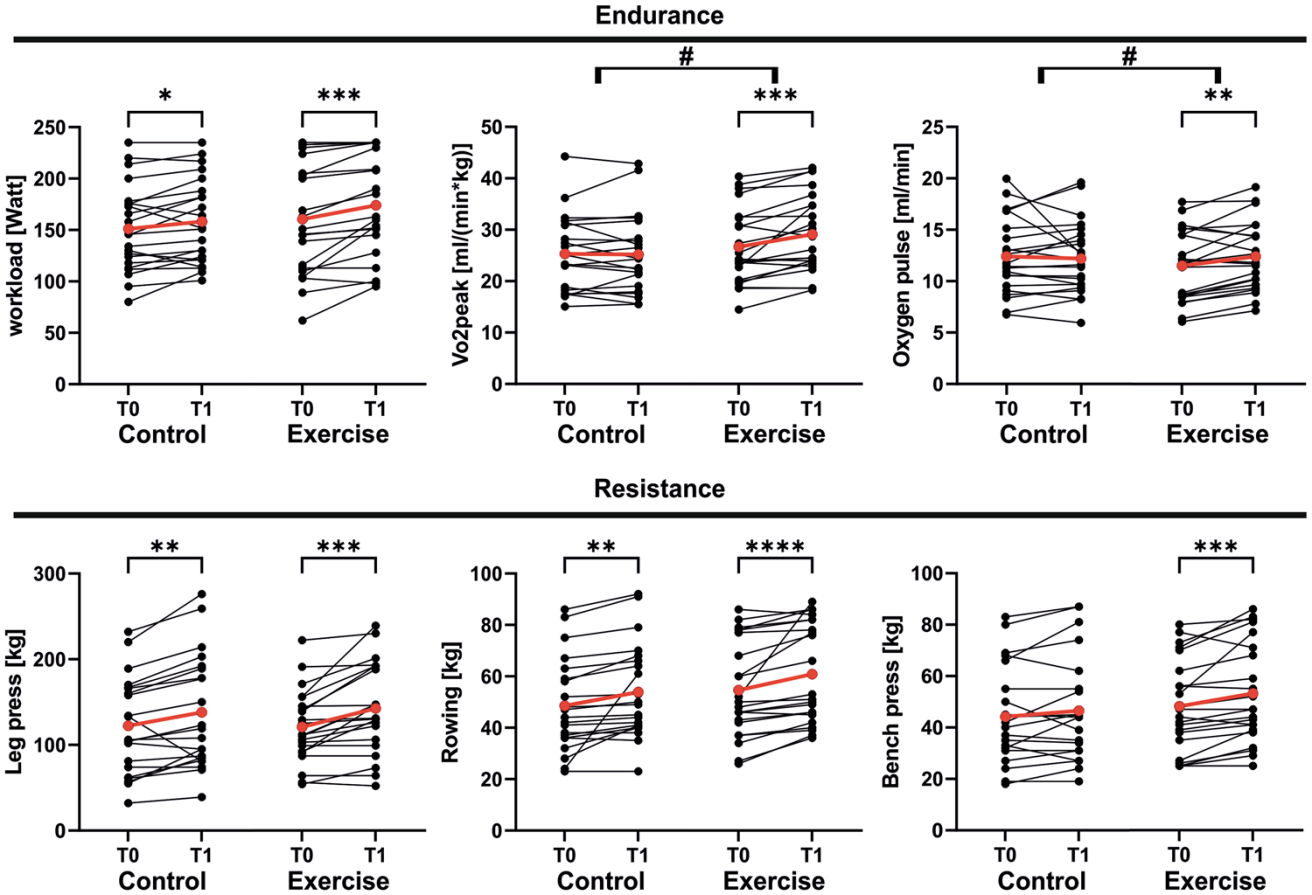
Changes in spiroergometric and strength parameters of the exercise and the control group between T0: baseline visit and T1: after exercise intervention or after the corresponding period in the control group. The red line represents the mean. *: *p < *0.05; **: *p < *0.01 ***: *p < *0.001, ****: *p < *0.0001, #: *p < *0.05 percentual change between groups

After 4 weeks of training, we found significant improvements in Ppeak 11.2 ± 15.1% (*p < *0.001), endurance capacity (VO2peak) 10 ± 12.7% (*p < *0.01), oxygen pulse 9.8 ± 10.8% (*p < *0.05) and strength in leg press: 18.2 ± 19.2% (*p < *0.001), rowing: 13.6 ± 16.7% (*p < *0.001) and bench press: 11.8 ± 13.5% (*p < *0.001) in the exercise group compared to baseline. The control showed only improvements compared to baseline in Ppeak 5.7 ± 10.7% (*p < *0.05), leg press 15.3 ± 20.6% (*p < *0.01), and rowing 15.3 ± 32.8% (*p < *0.01) (Table 3**, **Fig. 2). A group effect was found for VO2peak (*p < *0.01) and oxygen pulse (*p < *0.05) between exercise and control, respectively.

**Table 3:** Cardiopulmonary exercise test and strength test

		Exercise							Control							Group effects t1
		*t*0			*t*1			*p*	*t*0			*t*1			*p*	*p*
RCP																
Workload	Watt	111.8	±	33.5	120.6	±	39.2	0.082	110.1	±	31.9	112.7	±	33.8	0.609	0.570
Peak																
Workload	Watt	160.6	±	52.0	174.1	±	47.9	** < 0.001**	151.2	±	41.1	158.2	±	40.4	**0.025**	0.391
HR	bpm	166.3	±	18.3	166.3	±	16.9	0.988	151.7	±	25.4	153.0	±	25.3	0.775	0.092
RRsys	mmHg	179.8	±	29.3	184.8	±	24.2	0.714	178.0	±	13.9	181.9	±	21.0	0.504	0.323
RRdia	mmHg	90.7	±	21.5	95.4	±	17.1	0.562	92.9	±	13.2	91.0	±	9.3	0.468	0.154
VO2 peak	ml/min*kg	26.7	±	7.2	29.1	±	7.6	**0.001**	25.3	±	7.1	25.2	±	7.4	0.972	0.518
RER	ratio	1.16	±	0.07	1.16	±	0.08	0.940	1.15	±	0.11	1.18	±	0.11	0.159	0.602
VE	l/min	75.5	±	22.8	80.0	±	19.0	0.094	71.5	±	18.2	72.0	±	19.0	0.823	0.208
Respiratory rate	brpm	36.5	±	7.2	38.7	±	7.5	0.168	37.2	±	8.2	38.9	±	11.6	0.326	0.760
Oxygen pulse	(ml/min)/bpm	11.5	±	3.4	12.4	±	3.3	**0.003**	12.4	±	3.6	12.2	±	3.4	0.729	0.361
5 min recovery																
HR	bpm	111.1	±	14.4	106.9	±	13.1	0.109	103.9	±	20.5	100.5	±	17.7	0.215	0.234
Strength test (1-RPM)																
Leg press	kg	120.9	±	41.4	142.7	±	50.7	** < 0.001**	122.3	±	55.6	138.0	±	64.7	**0.003**	0.953
Rowing	kg	54.6	±	18.3	60.9	±	18.9	**0.001**	48.5	±	17.8	53.9	±	18.1	**0.006**	0.381
Bench press	kg	48.2	±	17.6	53.2	±	19.1	**0.001**	44.2	±	18.8	46.5	±	20.4	0.117	0.594

*HR* heart rate; *RRsys* systolic blood pressure; *RRdia* diastolic blood pressure; *VE* respiratory minute volume; *RCP* respiratory compensation point; *RER* respiratory exchange ratio

*p*-value < 0.05 are written bold

**Fig. 3:**
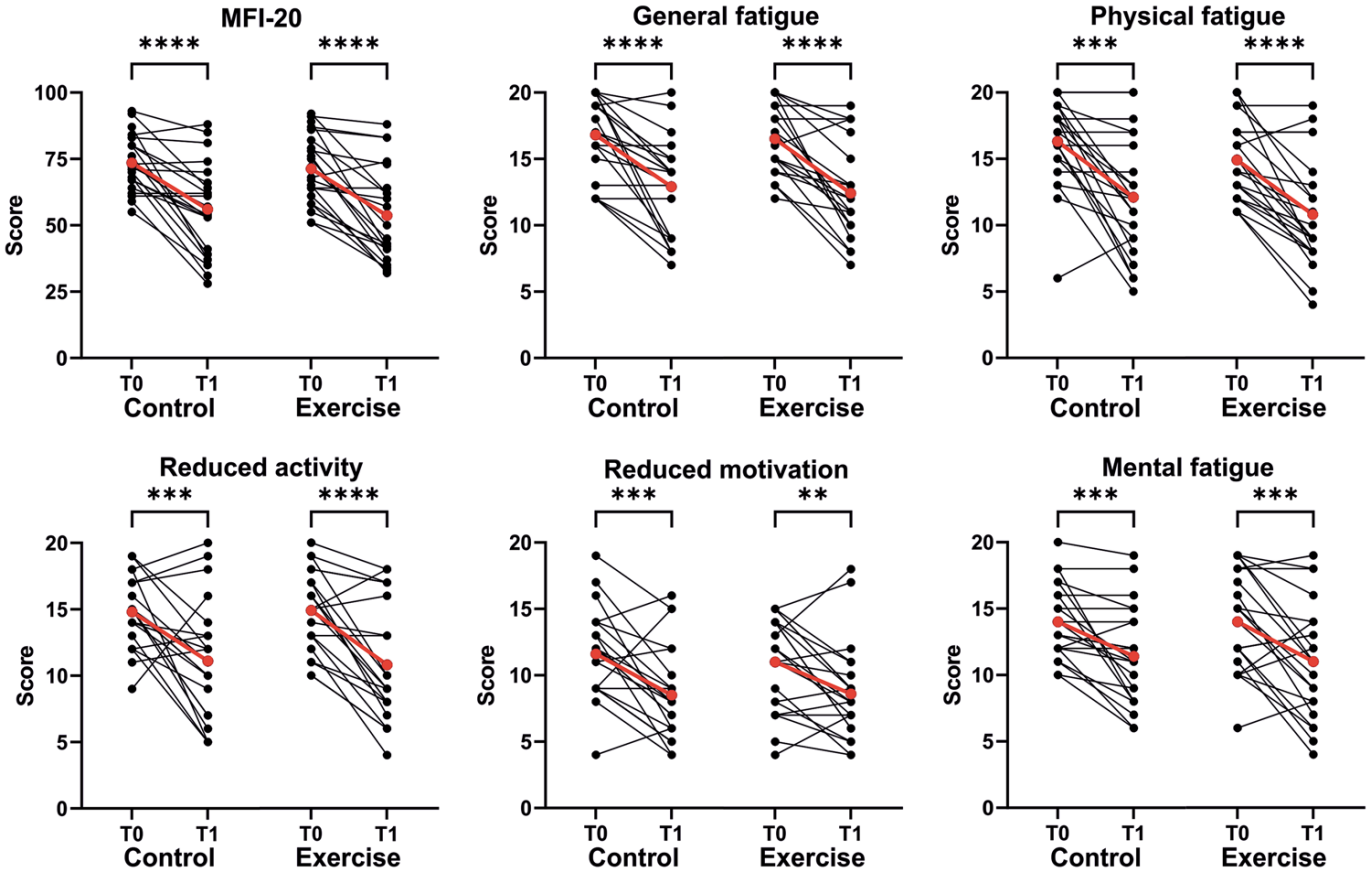
MFI-20 scores in sum and the five different dimensions. T0: baseline visit T1: after exercise intervention or after the corresponding period in the control group. The red line represents the mean. *: *p < *0.05; **: *p < *0.01 ***: *p < *0.001, ****: *p < *0.0001,

MFI-20 and quality of life (MQOL) were improved after 4 weeks in both groups. There was no difference in the MFI-20 score or the number of dimensions changed between the two groups (exercise − 2.2 ± 1.9 vs. control − 1.7 ± 1.7; *p = *0.428). The post-COVID-functional scale was also improved in both groups (exercise − 0.6 ± 0.9 vs. control − 0.5 ± 0.9; *p = *0.933) (Table 4**, **Fig. 3).

**Table 4:** MFI-20, MQOL, and PCFS

		Exercise				Time effect (t0 vs. t1)	Control				Time effect (t0 vs. t1)	Group effects t1	Group effects t2	Group effects t3
		t0	t1	t2	t3	p	t0	t1	t2	t3	p	p	p	p
MFI-20														
Sum	Score	71.3 ± 12.7	53.7 ± 17.4	55.3 ± 17.4	47.8 ± 19.8	** < 0.001**	73.5 ± 10.2	56.0 ± 17.0	58.8 ± 14.7	55.5 ± 16.4	** < 0.001**	0.927	0.636	0.336
General fatigue	Score	16.5 ± 2.5	12.4 ± 3.5	13.5 ± 3.8	11.9 ± 4.3	** < 0.001**	16.8 ± 2.9	12.9 ± 3.6	14.4 ± 3.2	13.7 ± 3.9	** < 0.001**	0.904	0.572	0.347
Physical fatigue	Score	14.9 ± 2.9	10.8 ± 4.0	10.7 ± 4.7	9.7 ± 5.1	** < 0.001**	16.3 ± 3.2	12.1 ± 4.4	12.9 ± 3.8	12.2 ± 3.7	** < 0.001**	0.434	0.178	0.144
Reduced activity	Score	14.9 ± 2.9	10.8 ± 4.4	11.4 ± 4.2	9.9 ± 4.5	** < 0.001**	14.8 ± 2.6	11.1 ± 4.3	11.6 ± 3.8	11.0 ± 4.0	**0.001**	0.370	0.938	0.647
Reduced motivation	Score	11.0 ± 3.4	8.6 ± 3.5	8.0 ± 3.2	6.8 ± 3.1	**0.006**	11.6 ± 3.3	8.5 ± 3.5	8.4 ± 3.1	8.3 ± 3.8	**0.001**	0.911	0.733	0.296
Mental fatigue	Score	14.0 ± 3.7	11.0 ± 4.2	11.7 ± 4.3	9.6 ± 4.2	**0.001**	14.0 ± 2.7	11.4 ± 3.6	11.4 ± 3.3	10.4 ± 3.5	**0.001**	0.623	0.690	0.547
MQOL														
Average	Score	21.2 ± 6.0	24.7 ± 5.7	25.2 ± 5.7	25.1 ± 6.8	** < 0.001**	18.3 ± 4.8	24.7 ± 5.7	23.0 ± 5.8	23.3 ± 6.0	** < 0.001**	0.836	0.235	0.074
PCFS														
Average	Score	2.6 ± 0.8	2.1 ± 1.1	2.2 ± 1.0	1.9 ± 1.1	**0.018**	2.8 ± 0.9	2.3 ± 0.9	2.2 ± 1.0	2.2 ± 1.0	**0.018**	0.412	1.000	0.547

*MFI-20* multidimensional Fatigue Inventory-20; *MGOL* McGILL Quality Of Life Questionnaire; *PCSF* the five point Post-COVID-Functional-Scale

*p*-value < 0.05 are written bold

**Fig. 4:**
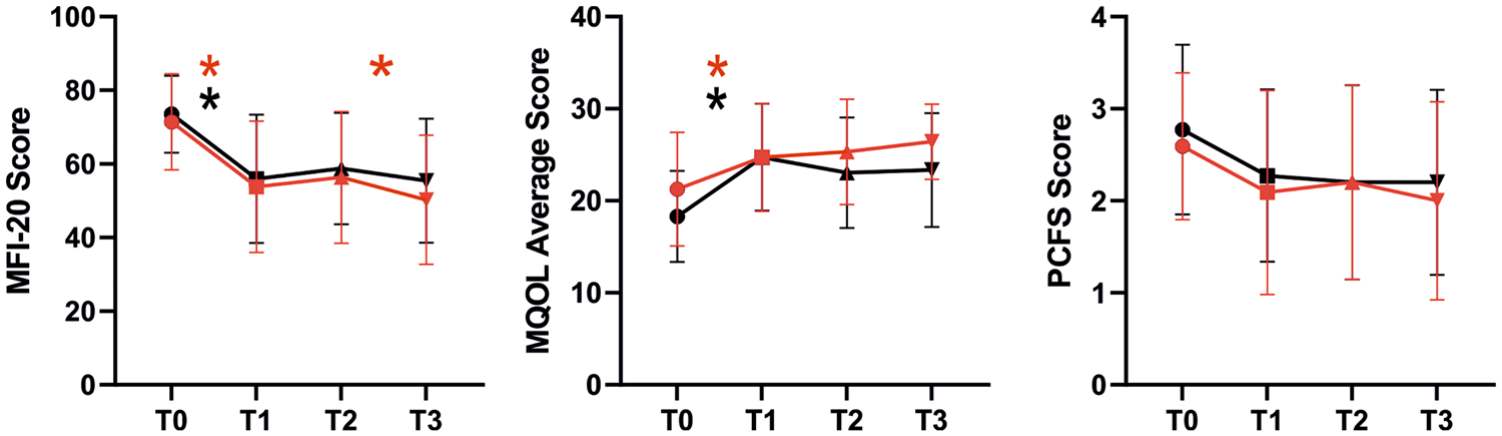
Follow-up questionnaires regarding fatigue and quality of life. T0: baseline visit T1: after exercise intervention or after the corresponding period in the control group T2: after 3 months, T3: after 6 months. The red lines represent the exercise group. *: *p < *0.05

After 3 months, there were no significant differences between the groups in any of the questionnaires or subdomains. After 6 months, the total physical activity per week was significantly greater in the exercise group than in the control group (exercise 1280 ± 1192 vs. control 644 ± 554, *p < *0.05). In addition, the subdomain of psychological quality of life in the MQOL was significantly better in the exercise group than in the control group (exercise 29 ± 9 vs. control 25 ± 9, *p < *0.05). Within the groups, only the MFI-20 of the exercise group improved significantly between the 3- and 6-month follow-up. The effects of the study remained stable through the follow-up period (Fig. 4). We saw no harm or unintended effects in our study.

## Discussion

The study shows that physical exercise is safe in patients with persisting symptoms post-COVID. Exercise improved endurance capacity, oxygen pulse, and strength compared to control. The self-reported quality of life and fatigue improved over time in both groups. In the follow-up period, the exercise group was more active and the MFI-20 improved more than in the control group. The physical training was not associated with additional effects on questionnaires or subdomains of fatigue compared to the patients randomized to the control group.

Our systematic search of the ongoing trials investigating exercise in post-COVID patients identified 107 records. Six records were removed due to duplication in different databases. Ultimately, 15 studies were found reporting a randomized design with an exercise intervention and a control group that underwent no intervention. Most of the studies (*n = *13) use a combined exercise intervention. An endurance-only intervention and a resistance-only intervention are planned in one trial each. The sample size, comparator types, blinding, and supervision of the ongoing RCTs are summarized in Supplemental Table 1. A detailed summary of the characteristics of each study can be found in Supplemental Tables 2–4. An overview of primary outcomes sorted into the groups defined by the potential mechanisms is shown in Supplemental Fig. 3. In comparison to other trials, our trial was the only one to use a supervised combined training regimen and follow-up for 5 months after the intervention ended. Although supervising exercise during an ongoing pandemic (as in our study) can be challenging, it is well established that supervised exercise yields a better result compared to unsupervised training programs [17]. Supervision also improves the safety of patients. Most studies identified by the systematic search of the literature use a combined training regimen. Combined exercise interventions target a wide spectrum of physiological dimensions of exercise capacity but conclusions about specific aspects of exercise that are effective are hard to draw. Combined exercise interventions may be more effective on submaximal exercise capacity and quality of life compared to isolated endurance exercise [18]. Therefore, a careful evaluation and studies of the effective training modality are needed to tailor a possible training intervention to the needs of patients post-COVID infection.

One major limitation compared to the other studies is the shorter duration of our training intervention.

The improvement in quality of life and fatigue post-COVID over time is supported by the literature [19]. Several potential limitations and explanations may contribute to the lack of an additional effect of physical exercise on fatigue: All training studies face a selection bias because only patients willing to participate in a training study can be randomized. These individuals may have a better prognosis reducing the additional effect of any intervention. Specifically, the subgroup of fatigue patients dominantly affected in the reduced motivation dimension of the MFI-20 may be under-represented. In our population, this dimension was the least affected.

Additionally, we found a higher daily activity in the exercise group in the follow-up period which is supportive of the under-representation of persons with reduced motivation. On the other hand, this greater activity can also be discussed as the cause of the improvement in the MFI-20 in the follow-up period.

The duration of the intervention may have been too short, however, we observed marked improvements in cardiovascular capacity in the intervention group. In one trial investigating exercise as a treatment option, the duration was 8 weeks compared to 4 weeks in our study. This study found effects in some fatigue-associated questionnaires, but the MFI-20 questionnaire was not used [19]. The sample size is too low for the identification of a subgroup potentially benefitting from exercise. However, there is no larger randomized training study in patients post-COVID published. The expected intra-group variability in physical performance, as well as fatigue symptoms at baseline, may be concealing effects. Another explanation may relate to the endpoint fatigue which is a very difficult-to-treat symptom. It is possible that the underlying pathologies of fatigue are not susceptible to physical exercise. Interestingly, fatigue does not correlate with the severity of the initial COVID infection, serum markers of inflammation or cardiovascular biomarkers, or cell turnover or echocardiographic findings [20, 21].

The symptoms post-COVID are characterized by physical fatigue, reduced activation, and impaired motivation. Our study shows that an individually planned and structured training regimen is highly effective in improving cardiovascular capacity, e.g. the maximum oxygen uptake and the oxygen pulse, in this patient population. Training effects have been consistently associated with a reduction of cardiovascular as well as all-cause mortality [22, 23].

In addition to the assessment of potentially positive effects, the safety of exercise post-COVID needs to be established. Physical exercise can promote sudden cardiac death in patients with myocarditis [24]. Sudden cardiac death has been reported in non-hospitalized patients with COVID-19 infection and mild symptoms [25]. However, dysrhythmias and cardiac arrest are rare in non-hospitalized individuals compared to hospitalized individuals with COVID-19 and also the presence of myocarditis in the athlete population post-COVID seems to be low [26]. Patients with persisting myocarditis should not be included in exercise programs according to international sport cardiology guidelines [27, 28]. Exercise aggravates myocarditis in animals [9]. Pro-arrhythmic effects of exercise in myocarditis are well established [29]. Myocarditis is a common cause of sudden cardiac death in athletes [10] and myocarditis is a frequent underlying cause of sudden cardiac death in physically active persons [30]. Our study, therefore, provides important reassurance that patients post-COVID without elevation of troponin and signs of cardiac diseases can safely perform physical exercise.

In conclusion, an individualized exercise program is a safe and well-tolerated treatment option in patients with persisting symptoms post-COVID. These patients significantly improve in physical performance after 4 weeks compared to patients without exercise intervention and these positive effects persist after the intervention ended. Exercise had no beneficial effect on fatigue symptoms or quality of life in our study. Additional results from longer or different exercise interventions on quality of life and fatigue are eagerly awaited.

### Supplementary Information

Below is the link to the electronic supplementary material.

Supplementary file1 (DOCX 1712 KB)

## Data Availability

The datasets generated during and/or analysed during the current study are available from the corresponding author upon reasonable request.
