# Supplementary material for: Physical exercise as a treatment for persisting symptoms post-COVID infection: review of ongoing studies and prospective randomized controlled training study
Source: Clin Res Cardiol. 2023 Sep 12;112(11):1699–709. doi: 10.1007/s00392-023-02300-6 (PMC10584711; doi:10.1007/s00392-023-02300-6)
Supplement: Supplementary file 1 — Supplementary file1 (DOCX 1712 KB) [file 392_2023_2300_MOESM1_ESM.docx]

**Supplements:**

**Supplemental Table 1 Systematic search of the literature – methodological features of the ongoing RCTs**

|  |  |  | Sample Size* | | Comparator Types | | Blinding | | Supervision of Exercise | | |
| --- | --- | --- | --- | --- | --- | --- | --- | --- | --- | --- | --- |
|  | **No. of Patients** | **No. of RCTs** | **<35** | **>35** | **Placebo attention group** | **No Inter-vention** | **Single** | **Double** | **None** | **Direct** | **Indirect** |
| Endurance Exercise | 90 | 1 |  | 1 | 1 |  | 1 |  |  | 1 |  |
| Resistance Exercise | 100 | 1 |  | 1 |  | 1 | 1 |  |  | 1 |  |
| Combined | 682 | 12 | 3 | 9 | 3 | 9 | 2 | 4 | 3 | 6 | 3 |
| Other | 60 | 1 |  | 1 | 1 |  |  | 1 |  | 1 |  |
| Total | 932 | 15 | 3 | 12 | 5 | 10 | 4 | 5 | 3 | 9 | 3 |

**Supplemental Table 2 Endurance intervention**

| **Study name** | **Inclusion criteria (abridged)** | **Sample Size** | **Study arms** | **Primary outcomes** | **Primary outcomes**  **Follow-up duration** |
| --- | --- | --- | --- | --- | --- |
| **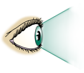COVID-CARE**  NCT04595773 | Age: 18 to 80 years  Post-COVID: > 0 days  Symptoms: n.n.  Other criteria:   - Total score ≤19 on the PROMIS short form for physical function or total score ≥9 on the PROMIS short form for fatigue, AND - Score ≥1 on the Patient Global Rating of Flu Severity and Patient Global Assessment of Interference with Daily Activities |  | 10 weeks x 3 sessions  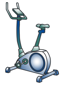  Aerobic exercise  (30min)  Vs.  10 weeks x 1 session  Education  (60min)  [Crossover design] | 6-MWT |  |

n.n.=not named, 6-MWT=6-minute walk test

**
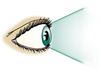
** supervised
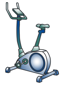
 endurance exercise

**Supplemental Table 3 Resistance intervention**

| **Study name** | **Inclusion criteria (abridged)** | **Sample Size** | **Study arms** | **Primary outcomes** | **Primary outcomes**  **Follow-up duration** |
| --- | --- | --- | --- | --- | --- |
| **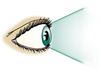NCT04797871** | Age: ≥ 18 years  Post-COVID: > 3 months  Symptoms: n.n.  Other criteria:   - There is no evidence on clinical records of pneumonia or any other organ failure related to SARS-CoV-2. |  | 12 weeks x 2 sessions  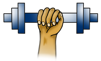  Resistance training   - 4 sets, - 8-12 repetitions, - 4 exercises - 50-75% 1RM   Vs.  No intervention | Changes in  lymphocytes numbers, monocytes number, monocyte expression |    |

1RM= one-repetition maximum, n.n.=not named

**
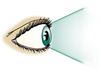
** supervised
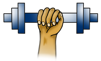
 resistance exercise

***Supplemental Table 4 Combined intervention / structured rehabilitation program / other***

| **Study name** | **Inclusion criteria (abridged)** | **Sample Size** | **Study arms** | **Primary outcomes** | **Primary outcomes**  **Follow-up duration** |
| --- | --- | --- | --- | --- | --- |
| **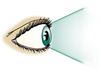COVID-Rehab**  NCT05035628 | Age: 50 to 80 years  Post-COVID: > 3 months  Symptoms: n.n.  Other criteria:   - presenting an increase of 1 point (compared to before infection) on the Modified Medical Research Council scale. |  | 8 weeks x 3 sessions  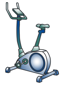 +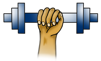  Aerobic exercise  (20min)  Resistance training  (20min)  Respiratory exercise  (10min)  vs.  No intervention | VO2max |  |
| **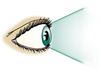**ACTRN12621000031864 | Age: ≥16 years  Post-COVID: > 3 months  Symptoms: any  Other criteria: n.n. |  | 8 weeks x 2 sessions  rehabilitation program via videoconferencing technology  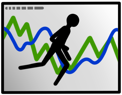   - Aerobic exercise - Resistance training - 60min   vs.  No intervention | 6-MWT |  |
| NCT05204511 | Age: ≥18 years  Post-COVID: > 3 months  Symptoms:   - one or more persistent symptoms that can be attributed to Post-COVID/Long-COVID - reduced physical performance capacity since infection   Other criteria: n.n. |  | 12 weeks x 3 sessions  Endurance training group:  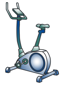  Aerobic exercise  (30-60min)  vs.  Combination Training group:  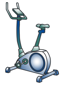 +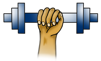  aerobic exercise (20-40 min)  Resistance training   - 2-3 sets, - leg press, - leg curls, - chest press and - seated horizontal rows   vs.  No intervention | VO2max |    |
| DRKS00026245 | Age: ≥18 years  Post-COVID: > 6 months  Symptoms:   - Performance impairment - Fatigue Assessment Scale (FAS) ≥ 22 points   Other criteria: n.n. |  | 12 weeks  Self-controlled/ telemedicine  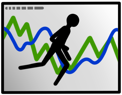   - daily activity, - aerobic exercises, - coordination, - relaxation, - stretching and - resistance training - total 150 min per week   vs.  No intervention | VO2max |      |
| NCT05119634 | Age: ≥18 years  Post-COVID: > 3 months  Symptoms: n.n.  Other criteria: n.n. |  | 8 weeks x 3 sessions  whole-body vibration training  vs.  home-based exercise program (not specified) | Short Physical Performance Battery  6-MWT  6-PBRT  IPAQ  activity monitoring with Actigraf GT3X |  |
| **RECOVERY**  NCT04958161 | Age: ≥ 55 years  Post-COVID: > 2 months  Symptoms:   - reduced exercise capacity, - fatigue, - cough, - shortness of breath, - headache and/or joint pain   Other criteria: n.n. |  | 12 weeks x 3 sessions  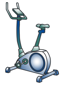 +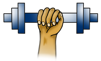  Aerobic exercise  (30min)   - 50-80% HRmax   Resistance training   - 1 set, - 8-12 repetitions, - 7 exercises   +  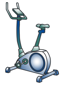  12 weeks × 2 sessions  home-based aerobic exercise  vs,  No intervention | 6-MWT SF-36 |  |
| **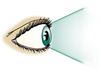**NCT05172206 | Age: ≥18 years  Post-COVID: > 3 months  Symptoms: any  Other criteria: n.n. |  | 3 weeks  Symptom-focused Rehabilitation  vs.  No intervention | Quality of life assessed by Short Form - 12 |    |
| RECOVE  **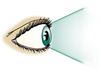**NCT04718506 | Age: ≥18 years  Post-COVID: > 3 months  Symptoms: any  Other criteria: n.n. |  | 8 weeks x 2 sessions  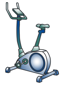 +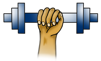  Aerobic exercise   - 4-6 × 3-5 min   (65-80% HRR)   - 2-3 min   (50-70% HRR)  Resistance training   - 2-4 sets, - 6-12 repetitions, - 5 exercises - 50-75% 1RM   +  8 weeks x 1 session  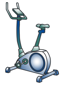  Aerobic exercise   - 30-60 min,   (65-70% HRR)  vs.  No intervention | Post-COVID Functional Status Scale |  |
| **COVIDPERS**  **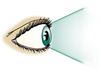**NCT04996212 | Age: ≥18 years  Post-COVID: > 3 months  Symptoms:   - dyspnea, OR - fatigue, OR - functional limitation.   Other criteria: n.n. |  | 8 weeks x 3 sessions  group 2  breathing exercises  group 3  combined   - breathing exercises with   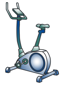   - aerobic exercises - 25-45 min   group 4  functional exercises   - breathing exercises with   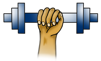   - Resistance training - 25-45 min   vs.  Control group  receiving a health education dossier | PostCovid-19 Functional Status Scale |    |
| **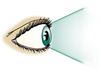**NCT04935437 | Age: ≥18 years  Post-COVID: ≥ 1 month  Symptoms:   - Reduction of physical activity, OR - Quality of life, OR - fatigue, OR - shortness of breath, OR - weakness of the upper and lower extremities, OR - post-traumatic stress, OR - pain, OR   Other criteria:  Priority will be given to patients suffering from more symptoms and disabilities.   - Patients admitted to the ICU, especially those who were intubated. - Patients who needed high oxygen mixtures (high-flow nasal cannula -HFNC, non-re-breathing mask)   older persons with prolonged hospitalization. |  | 8 weeks  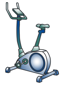 +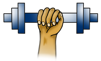   - aerobic exercise, - Resistance exercises, - physiotherapy, - psychological support and - dietary advice.   vs.  No intervention | Quality of life using SF-36  Depression  Cognitive dysfunction  Shortness of breath  Fatigue  6-MWT  … and more |  |
| NCT05003271 | Age: ≥18 years  Post-COVID: > 3 months  Symptoms:   - Mild to severe persistent respiratory symptoms   Other criteria: n.n. |  | 8 weeks x 3 sessions  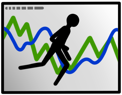  exercise program  in a zoom meeting (45 min)  vs.  exercise program while watching a pre-recorded YouTube video  (30 min) | lung capacity, dyspnea, fatigue, exercise capacity,  post-exercise O2-saturation, physical function (EQ-5D-SL),  activity participation,  SF-36 |  |
| **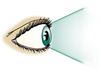**DRKS00026686 | Age: ≥18 years  Post-COVID: > 6 weeks  Symptoms:   - MFI-20: >50% (4 of 5 areas)   Other criteria: n.n.   - Normal LVEF - NTproBNP, Troponin-T (in normal range)   FEV1 and FVC within (in the normal range) |  | 4 weeks x 2-3 sessions  Combined training  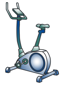 +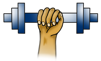  Aerobic training   - 2 x 4 min/ exercises - bike ergometer, - cross-trainer - 75-80% HRmax   Resistance training   - 2 sets, - 1 min /set repetitions, - 6 exercises - 70% 1RM   vs.  No intervention | Subjective improvement in perceived fatigue according to MFI-20 in at least 2 of 5 categories at follow-up |      |
| NCT04983394 | Age: ≥18 years  Post-COVID: > 3 months  Symptoms: any  Other criteria: n.n. |  | 8 weeks x 3 sessions  a motion-controlled video game with Microsoft Xbox One Kinect  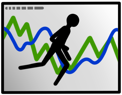   - aerobic exercises - Resistance training - stretching - 30 min   vs.  Combined training  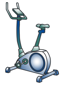 +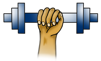   - aerobic exercises - Resistance training - stretching - 30 min | Pain on a visual Analogue Scale |  |

n.n.=not named, 6-MWT=6-minute walk test, VO2max =maximal respiratory oxygen uptake, HRmax=maximal heart rate, 6-PBRT=6 minutes pegboard and ring test, IPAQ= international physical activity questionnaire, MFI-20=multidimensional fatigue inventory-20 items,

**
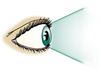
** supervised
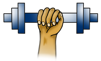
 resistance exercise


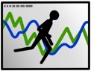
 telemonitored
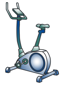
 endurance exercise

**Supplemental Figure 1 Potential mechanisms contributing to the post-COVID syndrome
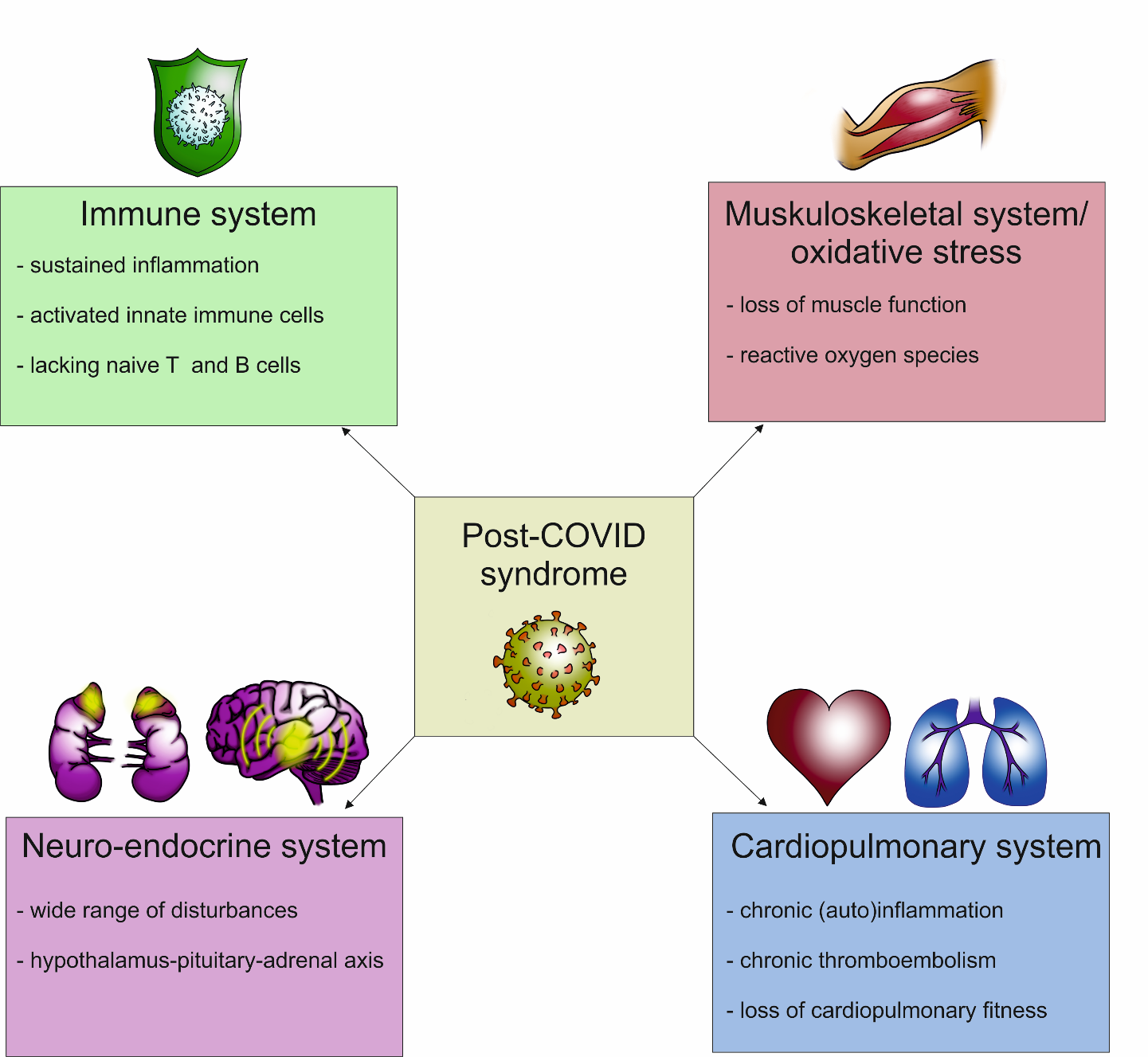
**

Supplemental Figure 2 Flow diagram of the search in the review process

**
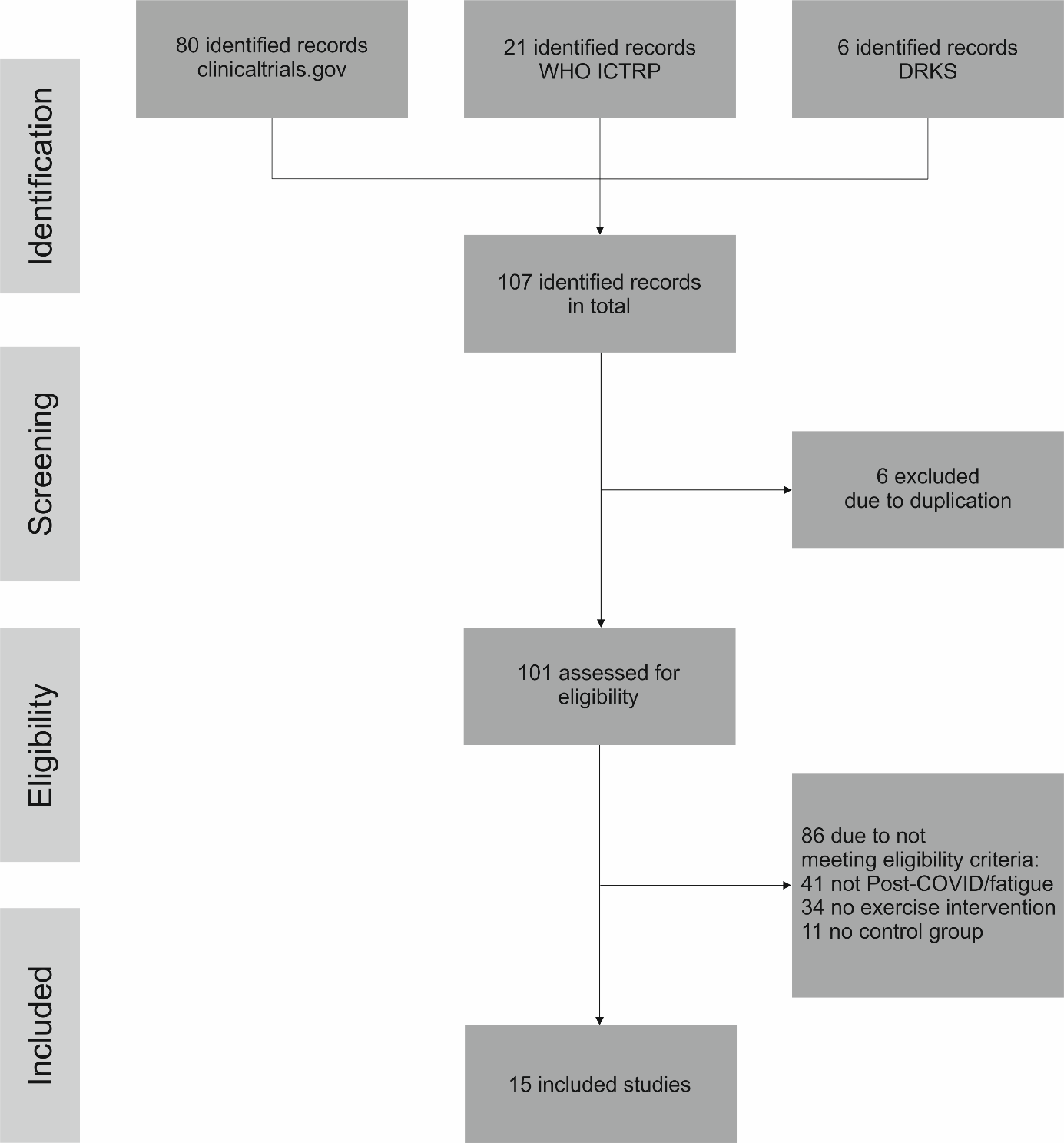
**

**Supplemental Figure 3 Parameters to assess the symptoms in individuals post-COVID infection**

**
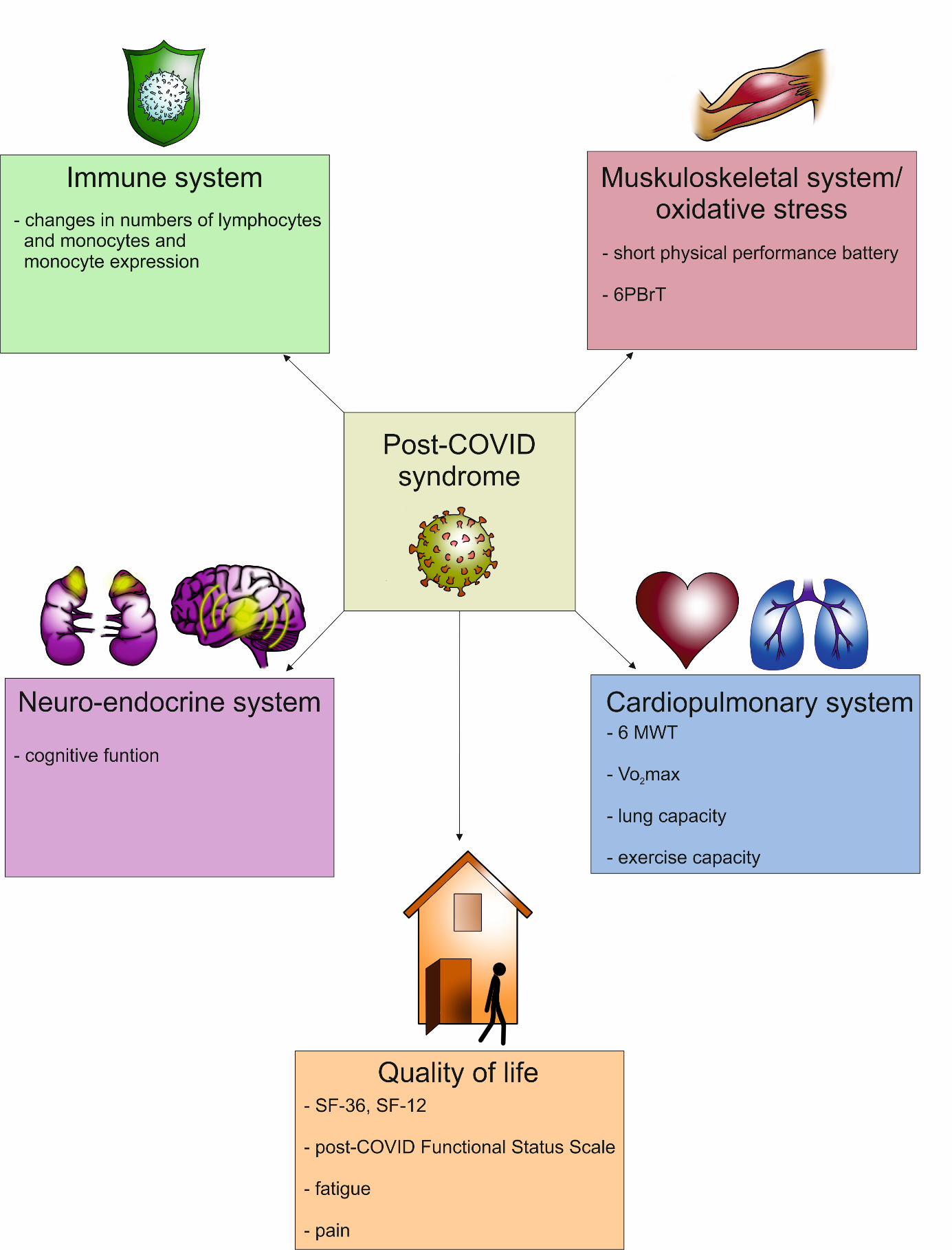
**
